# The PorX/PorY system is a virulence factor of *Porphyromonas gingivalis* and mediates the activation of the type IX secretion system

**DOI:** 10.1016/j.jbc.2021.100574

**Published:** 2021-03-21

**Authors:** Dezhi Yang, Chizhou Jiang, Bo Ning, Wei Kong, Yixin Shi

**Affiliations:** 1 The School of Life Sciences, Arizona State University, Tempe, Arizona, USA; 2 Biodesign Center for Immunotherapy, Vaccines and Virotherapy, Arizona State University, Tempe, Arizona, USA; 3 The Center for Cellular and Molecular Diagnostics, Tulane University School of Medicine, New Orleans, Louisiana, USA

**Keywords:** the two-component system, transcription regulation, the type IX secretion system, *Porphyromonas gingivalis*, virulence, the PorX/PorY system, HK, histidine kinase, qRT-PCR, quantitative real-time polymerase chain reaction analysis, RR, response regulator, T9SS, type IX secretion system, TCS, two-component system

## Abstract

PorX/PorY is a two-component system (TCS) of *Porphyromonas gingivalis* that governs transcription of numerous genes including those encoding a type IX secretion system (T9SS) for gingipain secretion and heme accumulation. Here, an *in vitro* analysis showed that the response regulator PorX specifically bound to two regions in the promoter of *porT*, a known PorX-regulated T9SS gene, thus demonstrating that PorX/PorY can directly regulate specific target genes. A truncated PorX protein containing the N-terminal receiver and effector domains retained a wild-type ability in both transcription regulation and heme accumulation, ruling out the role of the C-terminal ALP domain in gene regulation. The PorX/PorY system was the only TCS essential for heme accumulation and concomitantly responded to hemin to stimulate transcription of several known PorX-dependent genes in a concentration-dependent manner. We found that PorX/PorY activated the *sigH* gene, which encodes a sigma factor known for *P. gingivalis* adaptation to hydrogen peroxide (H_2_O_2_). Consistently, both Δ*porX* and Δ*sigH* mutants were susceptible to H_2_O_2_, suggesting a PorX/PorY-σ^H^ regulatory cascade to confer resistance to oxidative stress. Furthermore, the Δ*porX* mutant became susceptible to high hemin levels that could induce oxidative stress. Therefore, a possible reason why hemin activates PorX/PorY is to confer resistance to hemin-induced oxidative stress. We also demonstrated that PorX/PorY was essential for *P. gingivalis* virulence because the Δ*porX* mutant was avirulent in a mouse model. Specifically, this TCS was required for the repression of proinflammatory cytokines secreted by dendritic cells and T cells in the *P. gingivalis*–infected mice.

One of the most frequently occurring infectious diseases in humans is a chronic inflammatory disease of the periodontium that leads to erosion of the attachment apparatus and supporting the bone of teeth and is associated with increased risk for certain systemic disorders (1, 2, 3, 4, 5). Gram-negative anaerobic bacterium *Porphyromonas gingivalis* is the major causative agent of chronic periodontitis, which often colonizes in deep periodontal pockets of humans (6). On blood agar, *P. gingivalis* cells formed colonies with black pigment, which was derived from the protoheme in erythrocytes, and accumulated as a heme complex, *i.e.*, μ-oxo oligomers or dimeric heme comprising two Fe(III) protoporphyrin IX moieties bridged by an oxygen atom (7). *P. gingivalis* produces a broad array of virulence factors including cysteine proteinases (namely gingipains) for tissue colonization and destruction, as well as host defense perturbation (8, 9, 10, 11, 12). Two cysteine proteinase genes, *rgpA* and *kgp*, also encode hemagglutinins and the hemoglobin receptor at the 3’-terminal region of their transcripts (12, 13, 14). Gingipains were essential for the colony pigmentation of *P. gingivalis* (15) and secreted *via* a unique Por protein secretion system, now referred to as the type IX secretion system (T9SS) ((16, 17), also see a review (18)). T9SS plays an essential role in the accumulation of iron source and gingipain secretion since a mutant of the T9SS component gene, *porT*, exhibited beige colonies and could not secret gingipains (19).

Two-component systems (TCSs) provide the most ubiquitous signal transduction systems among a wide range of regulatory mechanisms developed in bacteria. Multiple TCSs ensure that bacteria can regulate transcription of a range of genes in response to a variety of environmental and intracellular stimuli and control an array of physiological traits, particularly bacterial virulence. Most TCSs consist of a sensor histidine kinase (HK), and a response regulator (RR), which usually functions as a DNA-binding protein. This design allows bacteria to directly sense and interact with a specific extracellular signal, which stimulates the kinase activity of the sensor protein to transfer a phosphoryl group to the cognate regulator, thus modulating transcription of particular genes. Although *P. gingivalis* seems to experience a number of environmental conditions in the oral cavity and therefore should monitor and respond to a variety of environmental cues, only four HK/RR pairs, one orphan HK, three orphan RRs, and one chimeric HK/RR systems have been characterized from different *P. gingivalis* genomes so far (20). Interestingly, a TCS identified in a *P. gingivalis* strain may not be functional in other strains. For example, PG0719 (HK) and PG0720 (RR) in a clinical isolate *P. gingivalis* W83 strain formed a TCS, HaeS-HaeR, which was activated by hemin to regulate genes for hemin acquisition including gingipains (21). However, the homologous region encoding this TCS in the less virulent 33277 strain contained PGN_0753 (RR), but carries a 2.5 kb deletion, causing a defective histidine kinase (PGN_0752) and a hemin-dependent growth phenotype (21).

The *porX* and *porY* genetic loci are annotated as *PGN_1019* and *PGN_2001* genes in 33277 strain and *PG0928* and *PG0052* genes in W83 strain, respectively. It has been demonstrated that these genes, which are located far apart on the *P. gingivalis* chromosomes, encode a TCS in which PorX is the RR, and PorY is the cognate sensor kinase (16, 22, 23). The PorX/PorY system was able to stimulate transcription of numerous genes, including many of those genes including *porK*, *porL*, *porM*, *porN*, *porP*, *porT*, and *sov* to encode the T9SS components (16). Consistently, a mutation at the *porX* locus, in a manner similar to disruption of T9SS, resulted in the accumulation of unprocessed gingipain proproteins, resulting in the reduction of Rgp and Kgp activities in bacterial cultures and a nonpigmented phenotype (24). It was once suggested that a chimeric HK-RR GppX also contributed to maturation and proper localization of gingipains to the outer membrane, thus influencing the activity of gingipains (25). Contrastingly, a recent study showed that both *gppX* deletion and insertion mutants retained the ability to exhibit black-pigmented colonies on the blood agar plates and produce wild-type-level gingipains (26). This similar phenotype between the wild-type strain and the *gppX* mutant ruled out the possibility that GppX played a role in modulation of the T9SS activity. It was demonstrated that the PorY and PorX proteins could interact with each other, and autophosphorylation of a truncated PorY protein and a subsequent phosphotransfer to PorX *in vitro* were stimulated by divalent cation Mn^2+^ (22). The PorX/PorY system was shown to regulate the T9SS-mediated protein secretion *via* an extracytoplasmic function sigma factor SigP, which was encoded by *PGN_0274* in 33277 strain (22, 27). However, another study reached a conclusion in contradiction to the first observation by showing that the PorX protein could only bind to the cytoplasmic domain of PorL subunit of the T9SS apparatus, but not directly bind to the promoters of the T9SS encoding genes (22, 23). Therefore, the molecular mechanism for how the PorX/PorY system participated in the regulation of *P. gingivalis* genes remained largely unclear.

*P. gingivalis* was able to interact with different types of host cells to manipulate immune response and finally escape killing from immune cells. For example, *P. gingivalis* infection modulated the release of chemokines and cytokines from fibroblasts (28). Particularly, *P. gingivalis* disarmed a host-protective pathway *via* proteasomal degradation of myeloid differentiation primary response 88 (MYD88), the key molecule in Toll-like receptor 2 (TLR2)-mediated immune responses (29). In addition, *P. gingivalis* secreted gingipains that inactivated several key proinflammatory mediators made by dendritic cells (DCs) and T cells by selectively inactivating most proinflammatory cytokines (30). By far, very little information is available on the contribution of TCSs to *P. gingivalis* virulence and signal transduction in response to the host environment. In this study, we established the role of the PorX/PorY system in the regulation of gene transcription and revealed the ability of PorX in recognition of the *porT* promoter. We also provided evidence that the PorX/PorY system is an essential regulator for *P. gingivalis* virulence.

## Results and discussion

### The *P. gingivalis* PorX/PorY system plays a major role in the accumulation of the iron source heme

**Figure 1:**
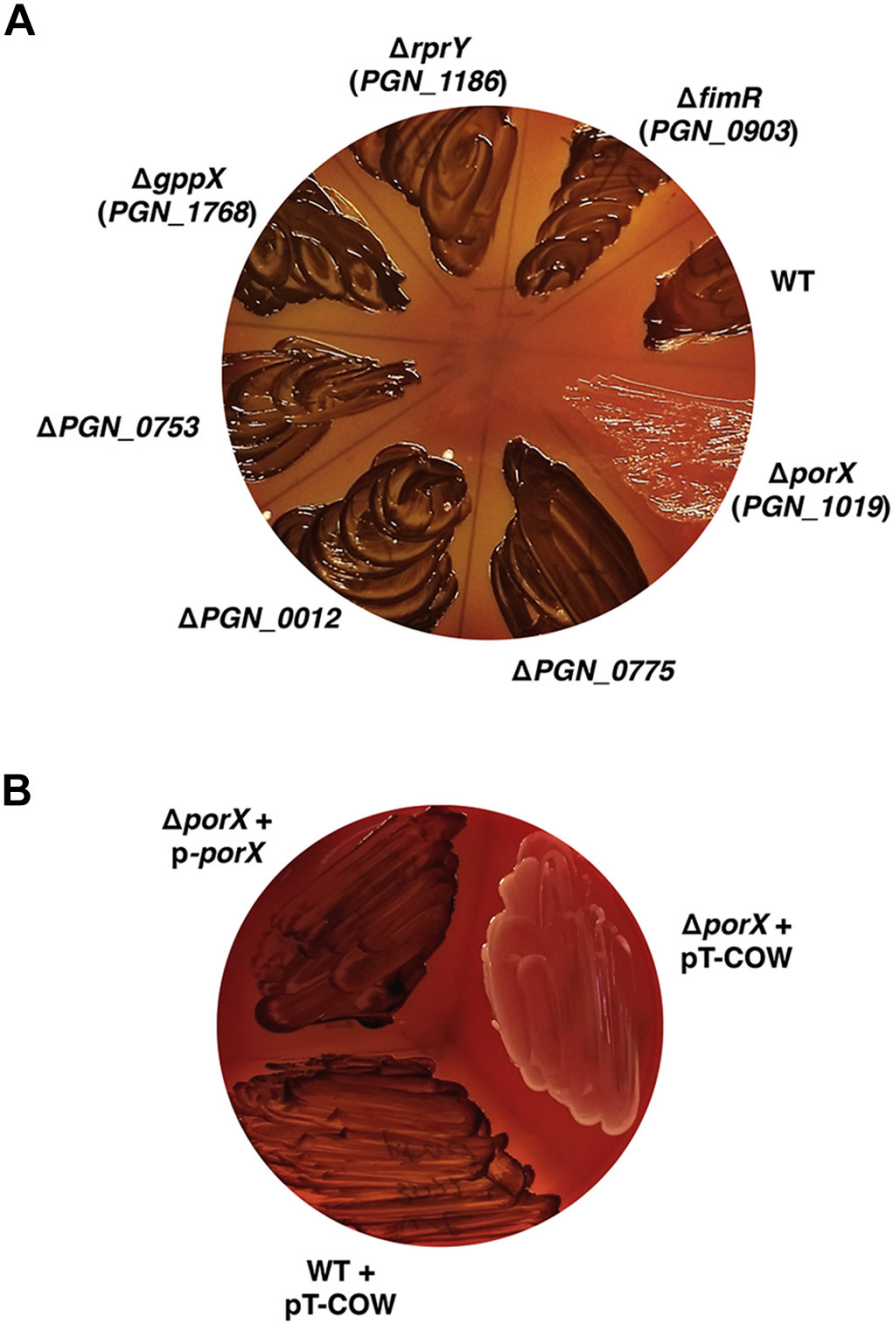
**The PorX/PorY system is the only two-component system essential for the accumulation of iron sources in *Porphyromonas gingivalis*.***A*, the growth of 33277 wild-type strain (WT), Δ*porX* (YS19181), Δ*PGN_0775* (YS18343), Δ*PGN_0012* (YS18345), Δ*PGN_0753* (YS17819), Δ*gppX* (YS18123), Δ*rprY* (YS18131), and Δ*fimR* (YS18069) mutants. *B*, the growth of 33277 WT with pT-COW (vector) and Δ*porX* mutant (YS19181) with pT-COW and the complementation plasmid pT-cow-P_*PGN_1016*_-*porX* (labeled as p-*porX*), respectively. All bacterial strains in (*A*) and (*B*) were grown anaerobically on sheep blood BHI agar plates at 37 °C for 7 days.

A *porX* null mutant of *P. gingivalis* 33277 strain displayed a nonpigmented phenotype due to disruption of T9SS, which subsequently reduced the accumulation of heme (24). We addressed the possibility of whether other TCSs could also influence the T9SS activity and then heme accumulation in *P. gingivalis*. Besides the *porX* gene, there were other six genetic loci encoding characterized and putative TCS RRs, *i.e.*, *PGN_0012*, *PGN_0753*, *PGN_0775*, *PGN_0903* (*i.e.*, *fimR*), *PGN_1186* (*rprY*), and *PGN_1768* (*gppX*) from a *P. gingivalis* 33277 genome (Uniprot, https://www.uniprot.org). We constructed single mutants in which one of the seven RR genes was disrupted by an erythromycin resistance cassette and observed their growth on a blood agar plate with enriched brain heart infusion medium (BHI). Consistent with the previous result (24), the *porX* deletion mutant (Δ*porX* mutant) produced a nonpigmented bacterial lawn (shown as beige, Fig. 1*A*). On the contrary, other regulator mutants retained the ability to form pigmented lawns as the wild-type strain (Fig. 1*A*), suggesting that these TCSs were not required for the heme accumulation. To further confirm that the nonpigmented phenotype was solely due to disruption of the *porX* gene, we introduced a complementary plasmid pT-COW-P_*PGN_1016*_-*porX* (referred to as p-*porX*, hereinafter) into the Δ*porX* mutant strain. In this bacterial cell, the PorX protein was produced *in trans* from p-*porX*, which carried the wild-type *porX* coding sequence controlled by the promoter region of the *porX* containing operon *PGN_1016*-*PGN_1021* (illustrated in Fig. S1). A wild-type phenotype was completely restored in this Δ*porX* mutant by p-*porX* because it was grown into a pigmented lawn like the wild-type strain while a Δ*porX* mutant carrying the parental plasmid pT-COW remained to form a beige lawn (Fig. 1*B*). This phenotypic analysis demonstrated that the PorX/PorY system was the major TCS essential for heme accumulation in *P. gingivalis*. Hence, it is reasonable to postulate that the PorX/PorY system is the major TCS for modulation of the T9SS activity.

### The receiver and effector domains of PorX are sufficient to confer its function for transcription regulation

**Figure 2:**
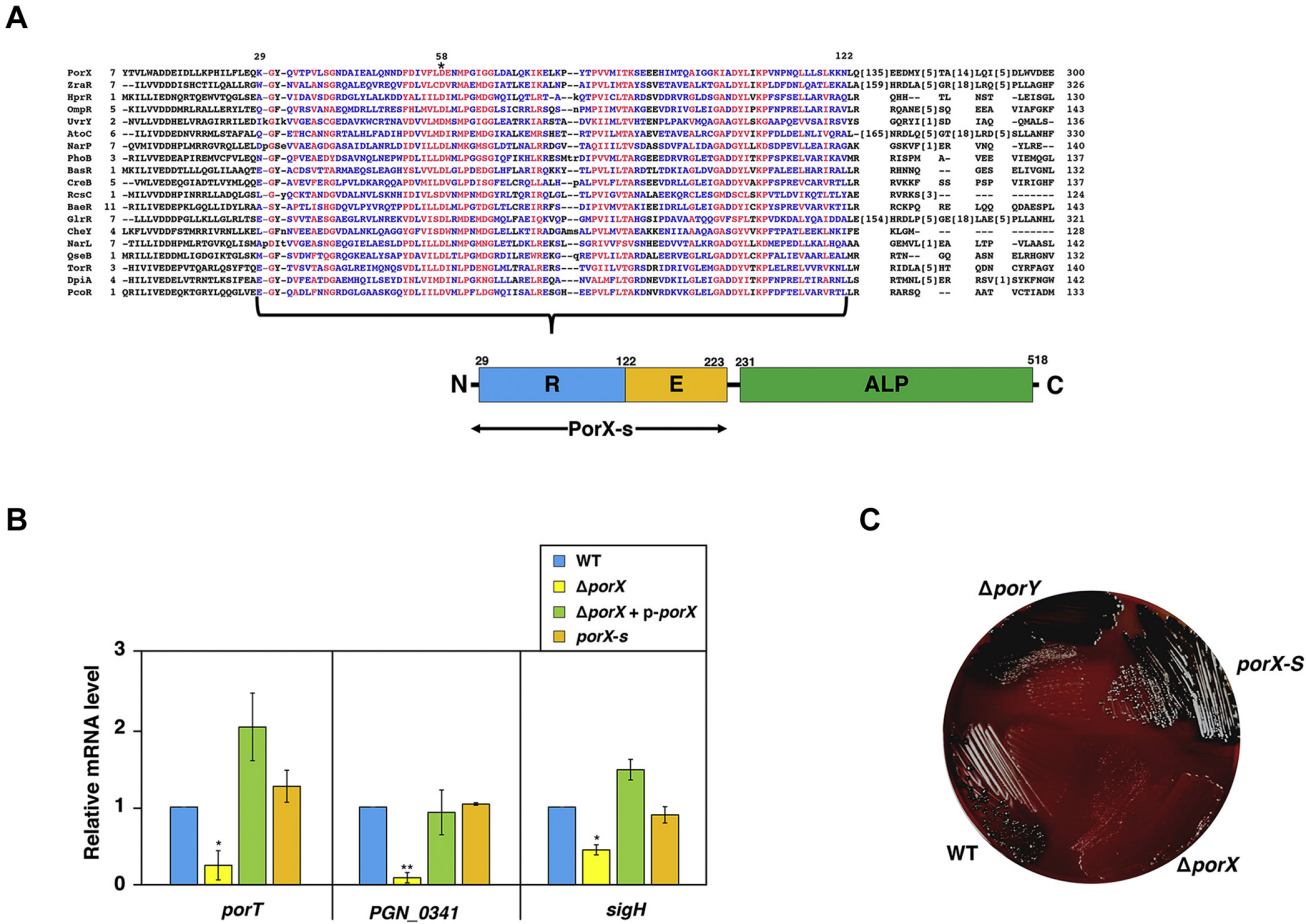
**The receiver and effector domains of the response regulator PorX are sufficient for gene regulation.***A*, a multiple sequence alignment of the N-terminal sequence of PorX with homologous sequences from TCS response regulators in *Escherichia coli* K-12. The *red color* indicates highly conserved columns and *blue* indicates less conserved ones. *Numbering* represents the positions of residues in individual proteins. The *bottom panel* is a model indicating the domains formed in the full-length PorX protein, in which R, E, and ALP represent the receiver domain, effector domain, and alkaline phosphatase-like core domain, respectively. The *double arrow* indicates the region included in PorX-s (the truncated PorX protein). *B*, qRT-PCR analysis of transcription of the PorX-activated *porT*, *PGN_0341* and *sigH* genes. The mRNA level was determined in 33277 wild-type strain (WT), Δ*porX* mutant (YS19181) with or without plasmid p-*porX*, and *porX-s* mutant (YS19363) grown in BHI for 48 h. The mRNA amount in WT was set to 1 for calculation of the relative mRNA level of the *porT*, *PGN_0341* and *sigH* genes in other strains. Results shown are representative of three independent experiments and all values were mean ± standard deviation and normalized to WT (fold change). ∗*p* < 0.05, ∗∗*p* < 0.01, *versus* wild-type, *t*-test. *C*, the growth of 33277 wild-type strain, Δ*porX* (YS19181), *porX-s* (YS19363), and Δ*porY* (YS19187) mutants grown anaerobically on sheep blood BHI agar plates at 37 °C for 7 days.

The *porX* gene product predicted as a 518-amino acid (aa) protein was shown to be phosphorylated by its cognate sensor kinase PorY (16, 22). According to a previous analysis (31), the C-terminal 231- to 518-aa region of this regulator formed an alkaline phosphatase-like core domain with a PglZ motif (ALP), which thus was unlikely to be directly involved in gene regulation. It has been well established that most TCS RRs possess a basic modular architecture built by a receiver domain (normally located in their N termini) with a conserved and phosphorylatable aspartate residue and a variable effector domain (in their C termini) to interact with the target promoters or specific cellular factors (32, 33, 34). Our result from a multiple sequence alignment (COBALT, https://www.ncbi.nlm.nih.gov/tools/cobalt/re_cobalt.cgi) revealed that the N-terminal 29- to 122-aa region of PorX shared similarities with the receiver domains of multiple RR proteins from Gram-negative bacterium *Escherichia coli* including those belonging to the OmpR/PhoB family (*Sequences with a brace* and *illustrated as R*, Fig. 2*A*). Concomitantly, its aspartate residue 58 (D58, *marked with an asterisk*, Fig. 2*A*) was equivalent to the aspartate residue conserved in all RR proteins, which had been shown to be phosphorylated by the cognate sensor kinases (35, 36). Based on these observations, we hypothesized that the first ∼223-aa sequence of PorX could form a modular architecture similar to those OmpR/PhoB-family RRs such as OmpR (239-aa), PhoB (229-aa), and BasR (222-aa) in *E. coli* (37, 38, 39). Consequently, the receiver domain (the 29- to 122-aa region) should coordinate with the downstream 123- to 223-aa region (*i.e.*, a receiver domain, *illustrated as E*, Fig. 2*A*) and form a functional structure to carry out transcription regulation. To investigate the regulatory activity conferred by this region, we constructed a 33277 strain (*porX-s*) in which a stop codon TAA (*arrowhead*, Fig. S2*A*) was added between the 223rd and 224th codons of the *porX* coding region through the insertion of a suicide plasmid (pGEM-*ermF*-*porX-s*, illustrated in Fig. S2*A*). This *porX-s* strain produced a truncated PorX protein (referred to as PorX-s), which contained the first 223-aa sequence, thus only carrying the receiver domain and the predicted effector domain (Fig. 2*A*, and Fig. S2*B*). A quantitative real-time polymerase chain reaction (PCR) analysis (qRT-PCR) was used to determine the transcription of two known PorX-activated genes, *i.e.*, *porT* (*PGN_0778*) and *PGN_0341* (16, 22), and the *sigH* (*PGN_1740*) gene, which was identified as a PorX-activated gene according to an RNA sequencing analysis and a proteomic analysis conducted by our laboratory recently (manuscript in preparation). The mRNA levels of *porT*, *PGN_0341*, and *sigH* in the wild-type strain were similar to those in the *porX-s* strain, but 3.9-, 10.4-, and 2.2-fold higher than those in the Δ*porX* mutant (Fig. 2*B*). The reduced mRNA levels of *porT*, *PGN_0341*, and *sigH* in Δ*porX* mutant were fully restored to wild-type levels by plasmid p-*porX* (Fig. 2*B*), confirming that the deficient expression of these loci indeed resulted from deletion of the *porX* gene. Our results demonstrated that the first 223-aa sequence still retained the PorX function in transcription regulation in a manner comparable to the full-length PorX protein. In support of this notion, the *porX-s* strain formed black-pigmented colonies on a blood BHI agar plate like its wild-type parental strain (Fig. 2*C*). Therefore, our observations clarified that the C-terminal ALP domain was not essential for PorX to control transcription regulation. Unlike the Δ*porX* mutant, a Δ*porY* mutant displayed as pigmented colonies on the blood BHI agar plate (Fig. 2*C*). In fact, a previous study had revealed that the contribution of PorX to gene regulation was greater than that of PorY (16). Thus, these observations implied that PorX might interact with a sensor kinase other than PorY to modulate gene regulation required for hemin accumulation in *P. gingivalis*.

### The PorX/PorY system is activated in a growth-dependent manner but not autoregulated

**Figure 3:**
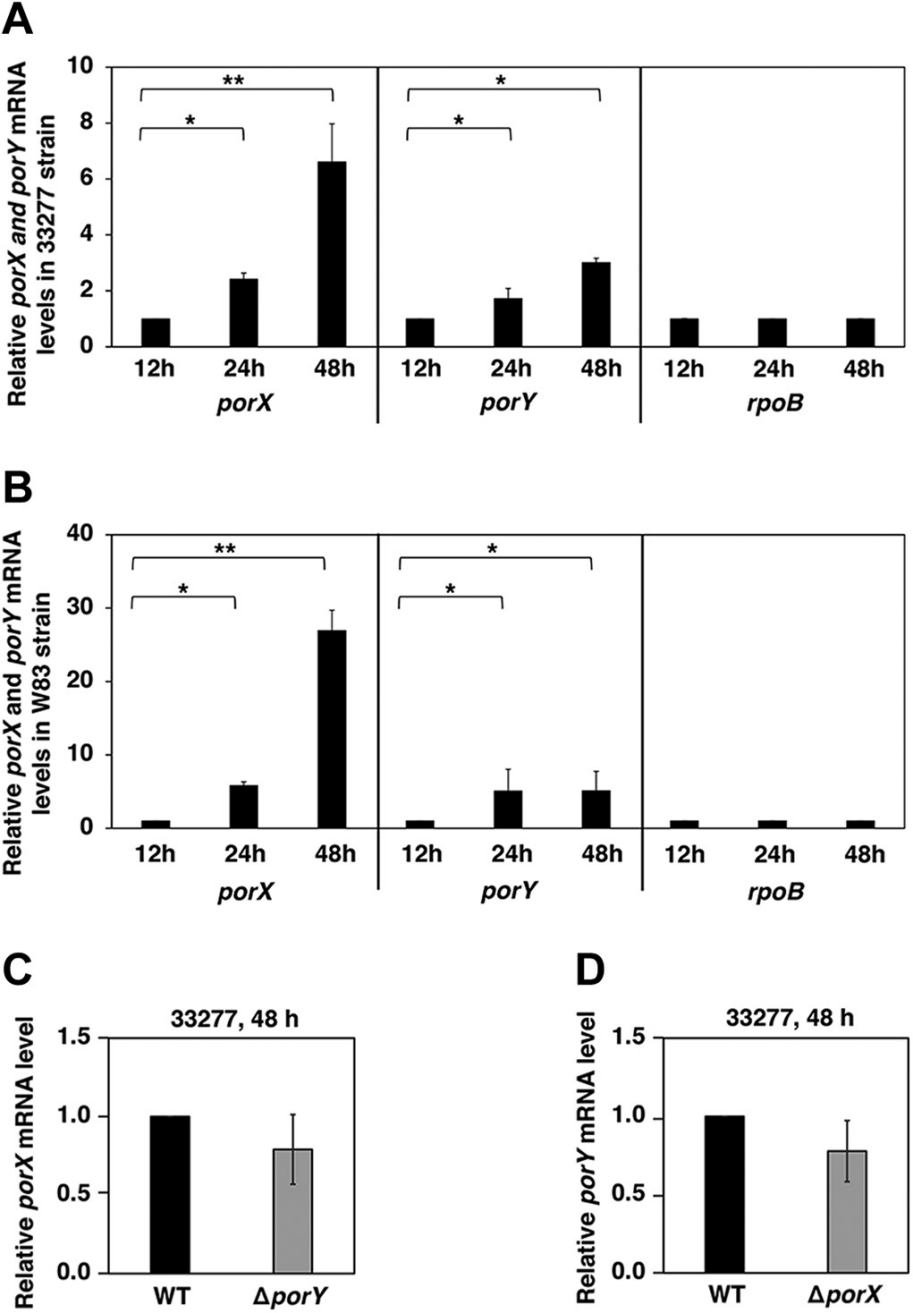
**The PorX/PorY system is not autoregulated.** qRT-PCR analysis of a time-dependent transcription of the *porX* and *porY* genes in 33277 wild-type strain (*A*) and W83 wild-type strain (*B*). The mRNA levels of the *porX* and *porY* genes were determined when bacterial cells were grown in BHI at the indicated time, and the mRNA amount of *rpoB* was set to 1 for calculation. *C*, the mRNA level of the *porX* gene was determined in 33277 wild-type strain and Δ*porY* mutant (YS19187) grown in BHI for 48 h. The mRNA amount from wild-type cells was set to 1 for calculation. *D*, the mRNA level of the *porY* gene was determined in 33277 wild-type strain and Δ*porX* mutant (YS19181) grown in BHI for 48 h. The mRNA amount from wild-type cells was set to 1 for calculation. Data correspond to three independent assays conducted in duplicate, and all values were mean ± standard deviation and normalized to the wild-type amount (fold change). The results shown are representative of three independent experiments. *p* > 0.05, *versus* wild-type, *t*-test.

We studied the expression of the PorX/PorY system in 33277 wild-type strain grown in BHI medium and found that mRNA levels of the *porX* and *porY* (*PGN_2001*) genes from the cultures of 24 and 48 h incubation were 6.6- and 3.0-fold higher than those of 12 h incubation, respectively (Fig. 3*A*). On the other hand, the mRNA level of the *PGN_1571*gene (*rpoB*), which encodes the RNA polymerase subunit beta subunit, remained constant at various growth times (Fig. 3*A*). Similarly, transcription of the *porX* and *porY* genes in a clinical isolate of *P. gingivalis*, W83 wild-type strain, was also stimulated significantly (26.9- and 5.1-fold, respectively) under the same condition (Fig. 3*B*). Therefore, the PorX/PorY system was continuously stimulated in a time-dependent manner during the *P. gingivalis* growth. Many TCSs were able to control the transcription of their regulator and sensor genes through autoregulation mechanisms (40). Thus, we investigated whether autoregulation was also responsible for activation of the *porX* and *porY* genes in spite of their locations far apart on the *P. gingivalis* chromosomes by comparing the mRNA levels of the *porX* and *porY* genes in the Δ*porX* mutant and the Δ*porY* mutant to those in the wild-type strain, respectively. The mRNA level of neither *porX* in the Δ*porY* mutant nor *porY* in the Δ*porX* mutant was significantly different compared with that of the wild-type strain grown in the BHI medium for 48 h (Fig. 3, *C* and *D*). These observations indicated that the PorX/PorY system was not autoregulated.

### The PorX/PorY system is stimulated by hemin and can protect *P. gingivalis* cells from overloaded iron source hemin

**Figure 4:**
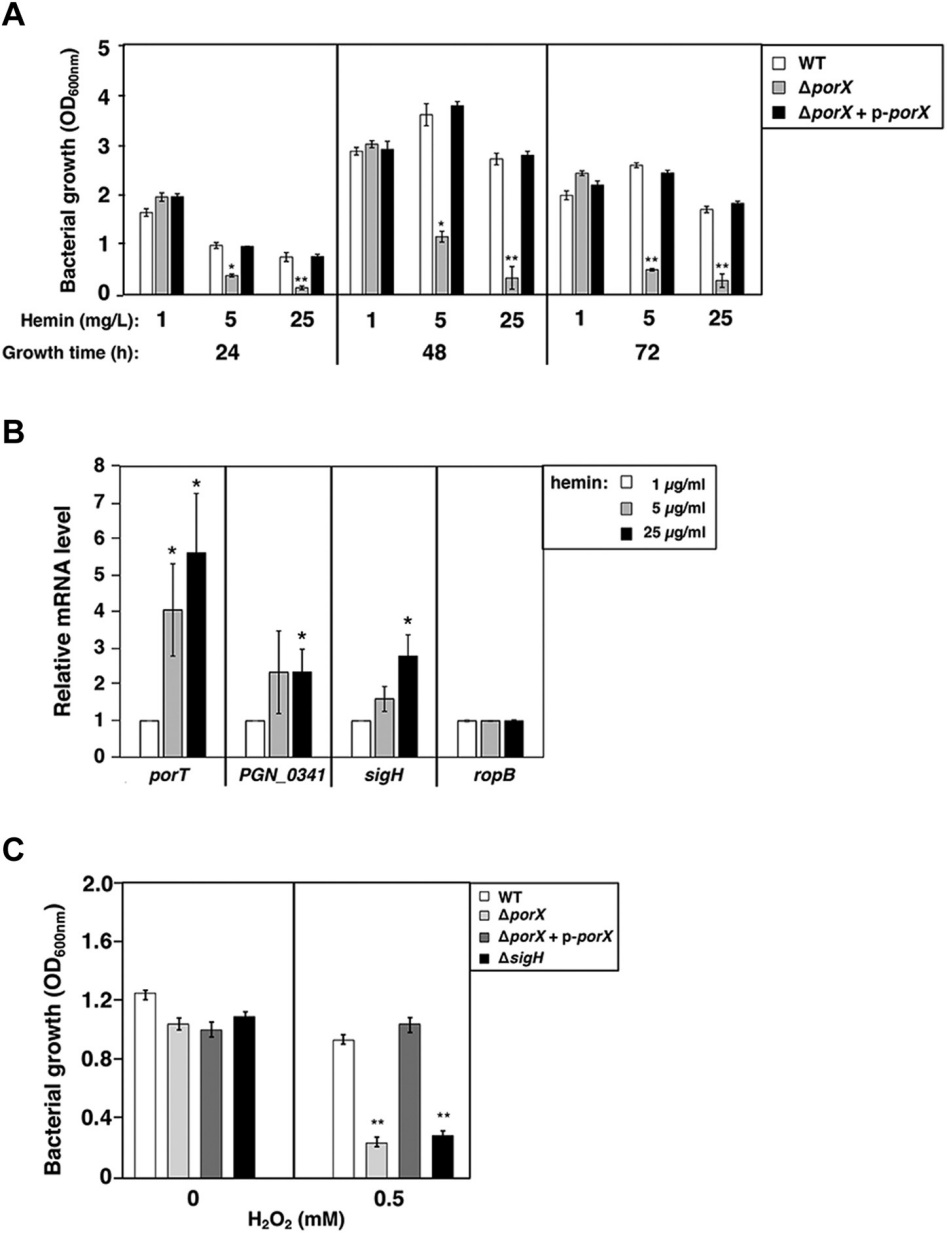
**The PorX/PorY system responds to hemin and confers resistance to H**_**2**_**O**_**2**_**.***A*, 33277 wild-type strain (WT) and Δ*porX* mutant (YS19181) with or without complementation plasmid p-*porX* (pT-COW-P_*PGN_1016*_-*porX*) were grown in BHI medium supplemented with indicated amounts of hemin for indicated times. Data correspond to three independent assays conducted in duplicate, and all values were mean ± standard deviation. The results shown are representative of three independent experiments. ∗*p* < 0.05, ∗∗*p* < 0.01, *versus* wild-type, *t*-test. *B*, qRT-PCR analysis of transcription of the PorX-activated *porT*, *PGN_0341*, and *sigH* genes in wild-type strain grown in BHI for 48 h. The *rpoB* gene was used as a negative control. The mRNA amount from bacterial cells grown with 1 μg/ml hemin was set to 1 for calculation of the relative mRNA levels in other cultures. Data correspond to three independent assays conducted in duplicate, and all values were mean ± standard deviation. ∗*p* < 0.05. *C*, 33277 wild-type strain, Δ*porX* (YS19181) with or without complementation plasmid p-*porX*, and Δ*sigH* (YS17717) mutants grown for 24 h were treated with 0.5 mM H_2_O_2_ and further incubated for 24 h. Data correspond to three independent assays conducted in duplicate, and all values were mean ± standard deviation. The results shown are representative of three independent experiments. ∗∗*p* < 0.01, *versus* wild-type, *t* test.

Although the PorX/PorY system contributed to the pigment phenotype of *P. gingivalis* for its utilization of the iron source (24), the underlying mechanism controlled by this regulatory system and associated physiological significance were not well known. We observed that the PorX/PorY system was important for *P. gingivalis* growth as hemin was the iron source. When bacterial cells were grown in BHI medium supplemented with 5 μg/ml hemin (*i.e.*, the recommended concentration) for 24, 48, and 72 h, respectively, the optical density at 600 nm (OD_600nm_) of the Δ*porX* mutant, was 2.6-, 3.1-, and 5.2-fold lower than that of the wild-type strain (Fig. 4*A*). Raising hemin concentration to 25 μg/ml (*i.e.*, five-time higher level) did not rescue, but further devastated, the deficient growth of the Δ*porX* mutant (Fig. 4*A*). This hemin-induced deficient growth was due to disruption of the PorX gene because OD_600nm_ of the Δ*porX* mutant cultured with both 5 μg/ml and 25 μg/ml of hemin was fully restored to the wild-type levels by plasmid p-*porX* (Fig. 4*A*). On the contrary, lowering hemin levels in BHI medium from 5 μg/ml to 1 μg/ml could reverse the deficient phenotype, allowing the Δ*porX* mutant to grow as well as wild-type strain (Fig. 4*A*). Based on these observations, we reasoned that hemin, particularly at high concentrations, exerted a harmful effect on the Δ*porX* mutant, which could be released by reducing its amount in the medium. Consistent with this notion, we observed that hemin overload was very toxic to the Δ*porX* mutant since many bacterial cells became unable to form colonies on a blood agar plate after being cultured in BHI medium with 25 μg/ml hemin for 48 h (data not shown). These results suggested that the PorX/PorY system should contribute to protecting *P. gingivalis* cells from a hemin overload instead of rescuing bacteria from hemin starvation. For this purpose, it was reasonable that the PorX/PorY system could respond to the hemin concentration, by which it could elicit gene regulation to rescue *P. gingivalis* cells from hemin overload. Indeed, the mRNA levels of *porT*, *PGN_0341*, and *sigH* in 33277 wild-type strain grown with 25 μg/ml hemin were 5.6-, 2.3-, and 2.8-fold higher than those grown with 1 μg/ml, respectively (Fig. 4*B*). This result demonstrated that hemin was able to enhance the transcription of the PorX/PorY-activated genes.

It was suggested that sigma factor SigH contributed to *P. gingivalis* resistance to oxidative stress induced by hydrogen peroxide (H_2_O_2_) (41). We postulated that the PorX/PorY system should be involved in this process because it actually activated transcription of the *sigH* gene (Fig. 2*B*). Consistent with this notion, results from a susceptibility assay showed that a *sigH* null mutant (Δ*sigH*) and the Δ*porX* mutant were 2.8- and 3.3-fold more susceptible to 0.5 mM H_2_O_2_ than the wild-type strain, respectively (Fig. 4*C*). The susceptibility of the Δ*porX* mutant to H_2_O_2_ was fully rescued by plasmid p-*porX* (Fig. 4*C*). Hemin had been suggested to be redox-active and could produce cytotoxic free radicals *via* peroxides to cause oxidative stress (42). We postulated that hemin overload should elicit an oxidative stress, which subsequently killed more susceptible Δ*porX* cells. Therefore, the PorX/PorY system must be a critical regulator to respond to iron source hemin and protect *P. gingivalis* from oxidative stresses. It remains to be determined whether the sensor PorY protein can directly bind a hemin molecule.

### Identification of the PorX binding site in the *porT* promoter region

**Figure 5:**
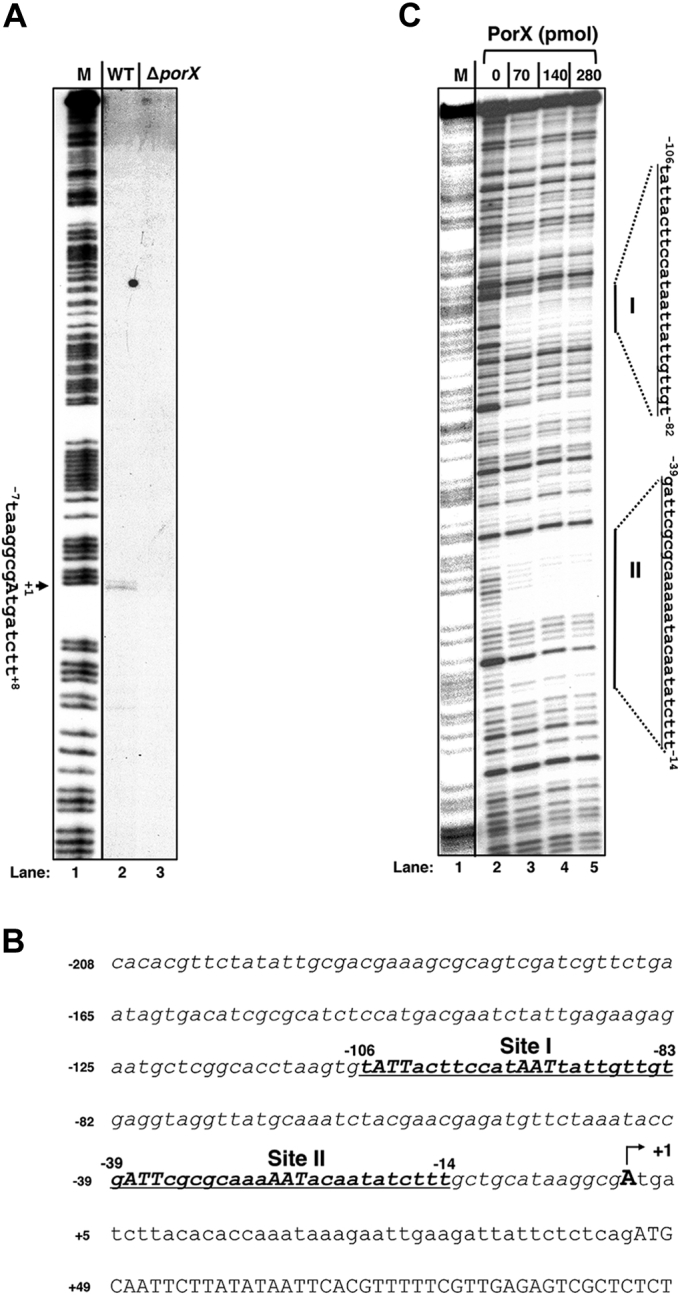
**The PorX response regulator binds to the *porT* promoter region *in vivo* and *in vitro*.***A*, mapping transcription start site of the *porT* gene in 33277 strains. Primer extension products from wild-type (WT, Lane 2 in the figure) and Δ*porX* (YS19145, Lane 3 in the figure) strains were generated using primer 4025 and total RNAs isolated from wild-type strain and Δ*porX* mutant (YS19181) grown in BHI medium for 48 h. *M* (Lane 1 in the figure) corresponds to a DNA ladder derived from a Maxam–Gilbert A + G reaction from a DNA fragment amplified with primers ^32^P-3043 & 3044. *Arrow* indicates the transcription start (*A*, also *labeled as +1*, also see next) representing 90th nucleotide from the 3’ end of the ladder. *B*, the DNA sequence of the *porT* promoter region. *Underline* corresponds to the PorX-protected regions (I and II) characterized below. *Underlined capital letters* represent the conserved nucleotides in both PorX-binding sites. *Numbering* begins from the transcription start site (+1) determined above and labeled with an *arrow*. *C*, DNase I footprinting analysis of the *porT* promoter fragment amplified with primers ^32^P-4025 & 4026 for the coding strand and increasing amounts of PorX-c-His_6_ protein (0, 70, 140, and 280 pmol, respectively, see Lanes 2–5 in the figure). Products were separated in polyacrylamide DNA sequencing electrophoresis, and the bands were detected by autoradiography. *Right lines* correspond to the PorX-protected DNA sequences and are labeled as I and II, respectively. The ladder *M* (Lane 1 in the figure) corresponds to the same ^32^P-labeled *porT* promoter fragment and degraded by the Maxam and Gilbert reaction.

A primer extension assay was conducted to map transcription initiation site of the *porT* gene, so that its full-length promoter sequence could be determined. A complementary DNA product was detected in a total RNA sample isolated from 33277 wild-type strain (Fig. 5*A*, *lane 2*), indicating that a *porT* transcription was initiated from the adenosine located in the 44-nt upstream of the open reading frame (illustrated in Fig. 5*B*, referred to as +1). The level of this product was dramatically reduced in the Δ*porX* mutant (Fig. 5*A*, *lane 3*), thus confirming that the transcription of the *porT* gene was upregulated by the PorX/PorY system. Then, we used a DNA fragment from upstream of the +1 site (the sequence covering the italic nucleotides, Fig. 5*B*) to investigate a direct interaction of the PorX protein and the *porT* promoter. An electrophoresis mobility shift assay (EMSA) showed that a PorX protein with a C-terminal His_6_ tag (PorX-c-His_6_) shifted this DNA fragment (Fig. S3), suggesting that this regulator should directly interact with the *porT* promoter. Therefore, we conducted a DNase I footprinting assay to characterize specific DNA regions recognized by PorX (*i.e.*, PorX binding site) in the *porT* promoter, and found that PorX-c-His_6_ protein protected two DNA sites from DNase I digestion (referred to as I and II, respectively, Fig. 5*C*). These two ∼25-bp sequences, *i.e.*, Site I, 5’-tattacttccataattattgttgtg-3’, and Site II, 5’-gattcgcgcaaaaatacaatatcttt-3’, were located in the −106 to −83 nt and −39 to −14 nt upstream of the +1, respectively (*underlined bold nucleotides*, Fig. 5*B*). Discovery of PorX-binding sites in the *porT* promoter verified that PorX can indeed control transcription directly, but not indirectly as suggested by a previous study (23). A conserved sequence, 5’-ATT-N_8_-AAT-3’, was observed from these two PorX-binding sites (*nucleotides shown as italic capital letters*, Fig. 5*B*). Therefore, in this study, we provided evidence that the PorX protein can directly bind to a PorX/PorY-dependent promoter and recognize specific sequences. So far, we have not been able to observe interactions of PorX to the *PGN_0341* and *sigH* promoters. It remains to be determined whether PorX/PorY regulated these genes indirectly. We predicted that a consensus DNA sequence recognized by PorX should be revealed by further biochemical analysis. It is worth noting that the PorX/PorY system has been shown to stimulate a sigma factor SigP, which thus binds to the promoter regions of T9SS component-encoding genes including *porT* and enhances their transcription (22). This observation and our results in this study suggest that this TCS should control the expression of T9SS *via* multiple regulators.

### The PorX/PorY system is essential for the virulence of *P. gingivalis* in a mouse model

**Figure 6:**
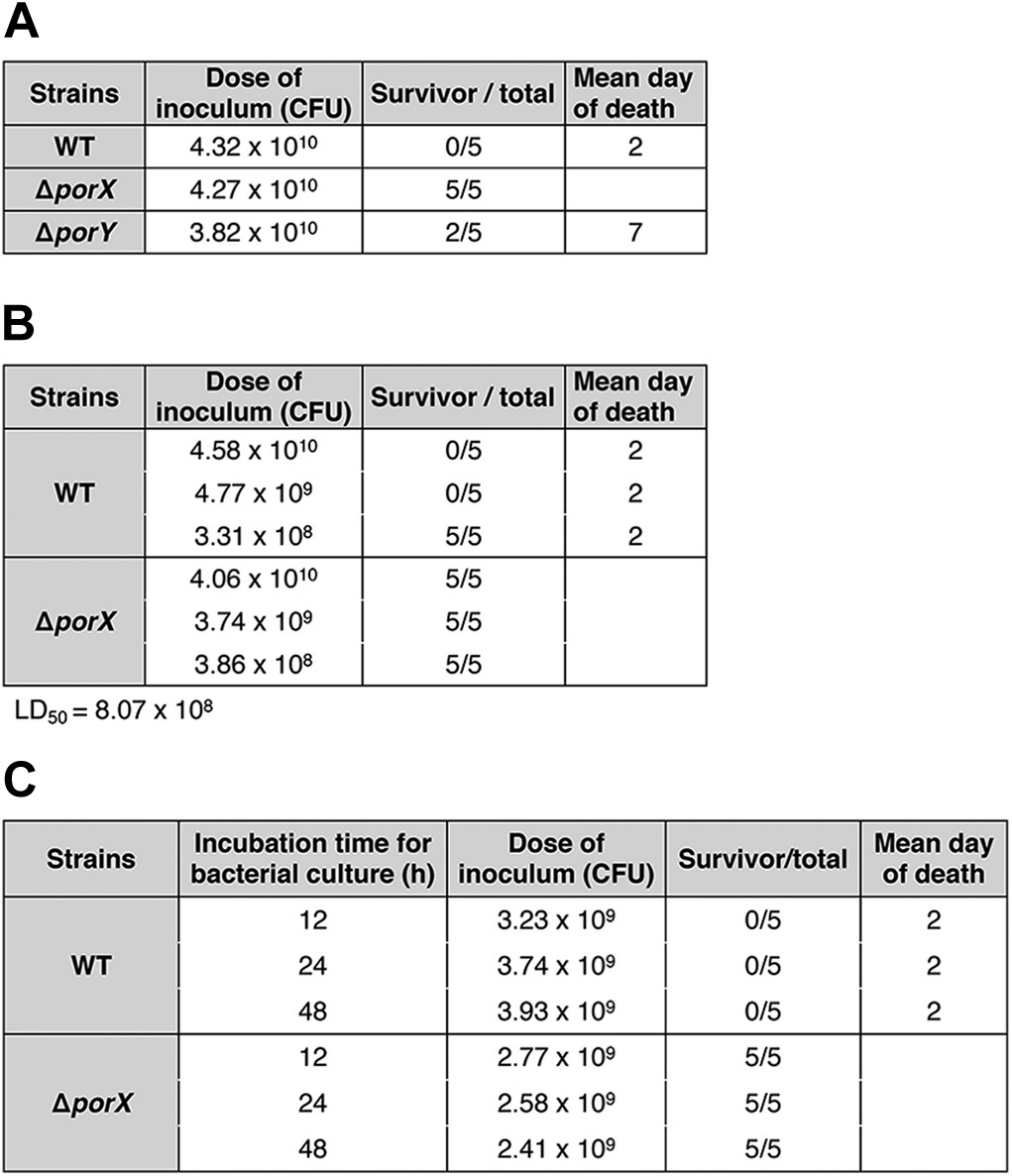
**The PorX/PorY-determined virulence of *P. gingivalis* W83 strains.***A*, virulence assay of *P. gingivalis* W83 wild-type, Δ*porX* (YS19145), and Δ*porY* (YS19153) strains. Groups of mice (*n* = 5) were inoculated with an indicated dose of *P. gingivalis* cells. *B*, LD_50_ of *P. gingivalis* W83. Groups of mice (*n* = 5) were inoculated with indicated doses of *P. gingivalis* cells. *C*, virulence assay of *P. gingivalis* W83 wild-type and Δ*porX* strains grown at different times. Groups of mice (*n* = 5) were inoculated with 2 to 4 × 10^9^ CFU of *P. gingivalis* cells.

The W83 strain has been used as a *P. gingivalis* strain for virulence testing in a mouse model (43). A previous study showed that all mice died by 48 h after challenge with W83 wild-type strain at a dose of 10^10^ CFU (44). We compared the PorX/PorY system in 33277 and W83 strains and found that they exerted similar effects on transcription regulation of the T9SS-encoding genes (manuscript in preparation). Hence, we evaluated the role of the PorX/PorY system in *P. gingivalis* virulence by comparing infection caused by W83 wild-type strain and the isogenic Δ*porX* and Δ*porY* mutants using this mouse model. Six-week-old BALB/c mice were subcutaneously injected on the dorsal surface with the strains grown in the BHI medium for 24 h, and all five mice died in 48 h after challenge with the wild-type cells at a dose of 4.32 × 10^10^ CFU (Fig. 6*A*). In contrast, all mice survived in the 30-day observation period after challenged with Δ*porX* mutant cells at a similar dose of 4.27 × 10^10^ CFU (Fig. 6*A*). This result demonstrated that the *porX* gene is an essential virulence factor because deletion of this locus rendered *P. gingivalis* avirulent. Unlike Δ*porX* mutant, the Δ*porY* mutant was attenuated but not avirulent since it still killed three out of the five mice at Day 7 postinoculation at a dose of 3.82 × 10^10^ CFU (Fig. 6*A*). This unequal contribution of PorX and PorY reinforced our assumption that PorX could interact with another sensor kinase to modulate gene regulation required for *P. gingivalis* pathogenesis.

The median lethal dose (LD_50_) of the W83 wild-type strain was determined by challenging mice with different doses of bacterial cells. We found that a dose similar to that used in Figure 6*A* (4.58 × 10^10^ CFU) and also a ∼10-time lower dose (4.77 × 10^9^ CFU) killed all five mice in 48 h, whereas all mice survived in the 30-day observation period after challenged with a ∼100-time lower dose (3.31 × 10^8^ CFU) (Fig. 6*B*). Therefore, the LD_50_ of the W83 wild-type strain should be 8.07 × 10^8^ CFU in this mouse model based on the formula previously described (45). Meanwhile, all mice challenged with the Δ*porX* mutant at similar doses survived (Fig. 6*B*), confirming that this mutant was avirulent. We also found that all mice were killed after challenged with ∼10^9^ CFU of the wild-type cells grown in BHI for 12, 24, and 48 h, respectively (Fig. 6*C*) although the PorX/PorY system was expressed at varying levels in these cultures (Fig. 3*B*). We concluded that the PorX/PorY system played a crucial role in the virulence of *P. gingivalis* in mice and also was highly sufficient to stimulate the expression of specific virulence factors for bacterial infection.

### The PorX/PorY system is critical for immune suppression induced by *P. gingivalis*

**Figure 7:**
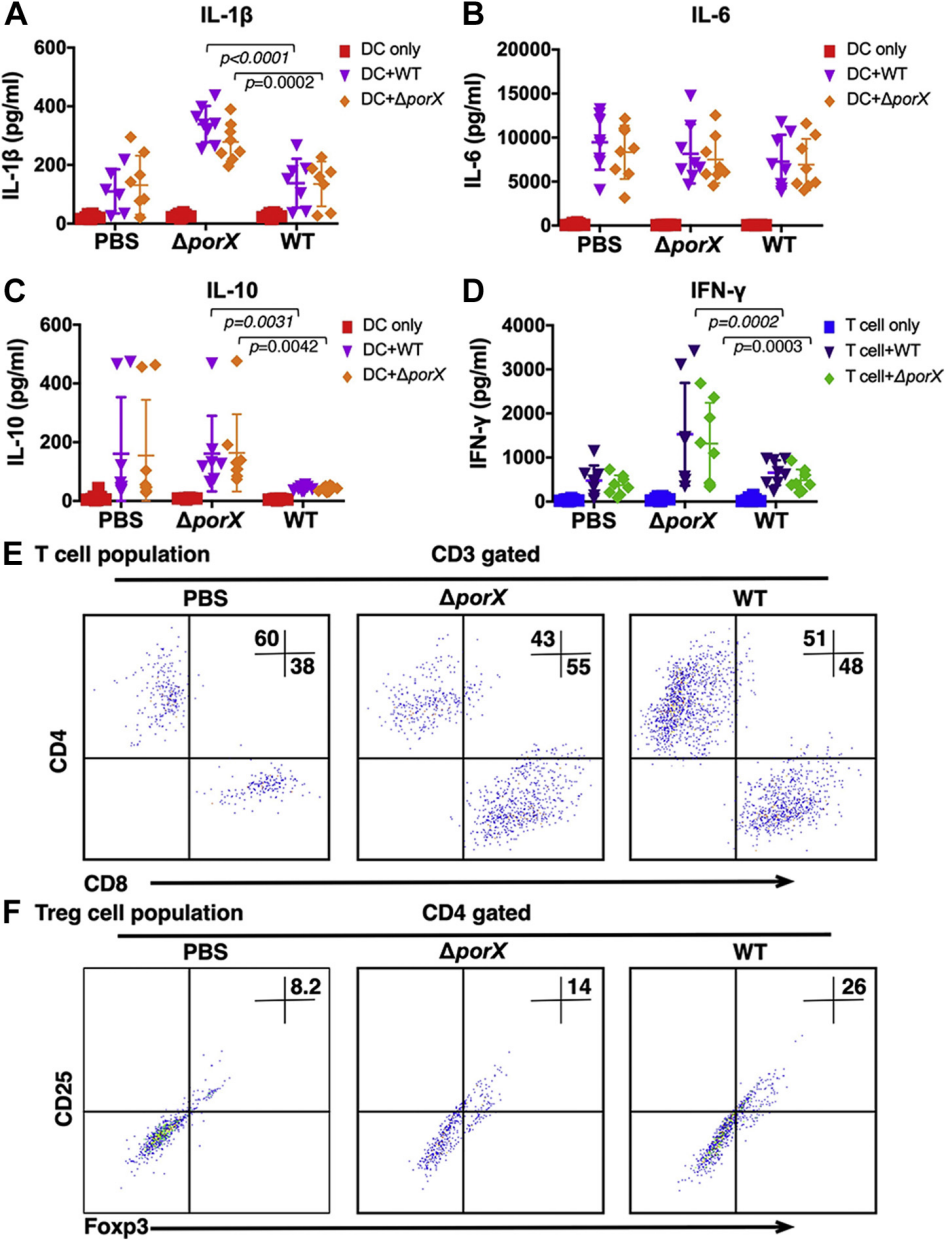
***P. gingivalis* immune responses in a mouse model.** DCs and T cells were isolated from mice either unstimulated (PBS) or stimulated on the 7 days with W83 wild-type strain or Δ*porX* mutant (YS19145) and cultured in the presence of wild-type strain or Δ*porX* mutant. In total, 25 μl of culture supernatants was collected, and IL-1β (*A*), IL-6 (*B*), and IL-10 (*C*) production from DC and IFN-γ production from T cells (*D*) were analyzed by Luminex Magpix multiplexing system. These data are expressed as dot plot with the mean ± SD (n = 8 mice). *p*-value was calculated using a one-way ANOVA (WT group *versus* Δ*porX* group). Cells were harvested, stained with directly labeled monoclonal antibodies, and analyzed by FACS analysis. CD4+ and CD8+ T cell populations (*E*) and CD25+/Foxp3+ regulatory T (Treg) cells (*F*) were analyzed by flow cytometry.

Previous studies have shown that most proinflammatory cytokines secreted by dendritic cells (DCs) and T cells are selectively inactivated in the mice infected with *P. gingivalis* W83 wild-type strain (30). Thus, inactivation of the mouse immune system might contribute to *P. gingivalis*-caused lethal, yet the detailed mechanisms remain unclear. To reveal the role of the PorX/PorY system in manipulating mouse immune cells, we isolated DCs and T cells from mice infected with a sublethal dose (5 × 10^8^ CFU per mouse) of W83 wild-type strain or Δ*porX* mutant and evaluated the cytokine release from these cells. We found that infection with the Δ*porX* mutant induced mouse DCs to release proinflammatory cytokine interleukin 1β (IL-1β) twofold higher than that with the wild-type strain (Fig. 7*A*), but not IL-6 (Fig. 7*B*). Interestingly, IL-10 expression significantly decreased in wild-type-infected mouse but recovered in Δ*porX* mutant-infected mouse (Fig. 7*C*), indicating that the death might be caused by an uncontrolled immune response. In T cell responses, the Δ*porX* mutant also induced a higher level of T cell activation associated with 2.3-fold increased interferon γ (IFN-γ) secretion (Fig. 7*D*). In another aspect, *P. gingivalis* infection increased CD8^+^ T cell 17% in Δ*porX* mutant-infected mice and 10% in wild-type-infected mice (Fig. 7*E*). The regulatory T cell (Treg) population in the mice infected by Δ*porX* mutant was 12% lower than those infected by wild-type strain (Fig. 7*F*). Taken together, our results showed that Δ*porX* mutation of *P. gingivalis* reversed immune repression in mouse DCs and T cells, revealing a critical role of the PorX/PorY system in *P. gingivalis*–induced immune responses.

## Concluding remarks

Our findings have led to an understanding of the following fundamental characteristics of the PorX/PorY system that confers gene regulation required for *P. gingivalis* virulence. (i) The PorX/PorY system actually functions as a typical TCS. In this study, we provided *in vivo* and *in vitro* evidence that RR PorX recognizes and binds to two DNA sequences in the *porT* gene (Fig. 5, *A* and *C*), which thus deciphered the molecular mechanism for the PorX/PorY-controlled expression of T9SS. We further demonstrate that the N-terminal receiver and effector domains are sufficient for PorX to confer its regulatory activity to gene transcription, which subsequently supports the notion that the C-terminal ALP domain is dispensable for transcription regulation. This ALP domain may possess an alternative function, perhaps to interact with the T9SS complex as suggested previously (23). (ii) Transcription of the T9SS component-encoding genes should be coordinately regulated by multiple regulators. Our foot-printing results and a previous study (22) suggested that both PorX and a PorX/PorY-stimulated sigma factor SigP should be able to directly interact with the *porT* promoter. It is reasonable that these two factors bind to specific promoter regions to initiate *porT* transcription. (iii) The PorX/PorY system can respond to iron source hemin and confer *P. gingivalis* resistance to oxidative stress. We demonstrate that hemin can stimulate PorX/PorY-regulated genes including *porT*, *PGN_0341*, and *sigH* in a concentration-dependent manner (Fig. 4*B*). Concomitantly, the Δ*porX* mutant grows very poor in high hemin concentrations and becomes more sensitive to H_2_O_2_ (Fig. 4, *A* and *C*). We also show that the PorX/PorY system contributes to activation of SigH, *i.e.*, a sigma factor required for adaptation to oxidative stress (46), which further validates the role of this TCS in *P. gingivalis* resistance to H_2_O_2_. Hence, the response of the PorX/PorY system to hemin must be beneficial for *P. gingivalis* cells to release oxidative stress, which is elicited by a hemin overload. (iv) The PorX/PorY system is a crucial regulatory system contributing to *P. gingivalis* virulence (Fig. 6, *A*–*C*). Furthermore, results from a mouse model provide evidence that *P. gingivalis* has developed the PorX/PorY system to conduct immune suppression during infection.

## Experimental procedures

### Bacterial strains, plasmids, medium, and growth conditions

Strains and plasmids used in this study are listed in Table 1. The *P. gingivalis* ATCC 33277 and W83 wild-type strains used in this study were obtained from Dr Koji Nakayama (16). *P. gingivalis* cells were grown at 37 °C in an anaerobic chamber (Model 2000, Coy Lab Products) that maintained 90% N_2_/5% CO_2_/5% H_2_ in the atmosphere. Blood agar plates (5% sheep defibrinated blood, and 1.5% agar) or Brain Heart Infusion (BHI, purchased from BD) medium supplemented with hemin (5 μg/ml, or a concentration required for bacterial growth when necessary) was used to culture *P. gingivalis* strains. When necessary, erythromycin (0.5 μg/ml) was supplemented in the medium. *P. gingivalis* cells were harvested by centrifuging liquid cultures at 10,000*g* (∼8500 rpm) for 10 min in a Sorvall ST 8R centrifuge with HIGHConic III Fixed Angle Rotor (Max. 9500 rpm) at 4 °C. *E. coli* DH5*α* and BL21 (DE3) strains were used for cloning and protein production, respectively. *E. coli* cells were routinely grown in Luria Broth (LB) supplemented with antibiotics when necessary (kanamycin, 50 μg/ml; ampicillin, 50 μg/ml) at 37 °C. To prepare cell lysates, bacterial cells were opened with a sonicator (Misonix Sonicator 3000).

**Table 1:** Bacterial strains used in this study

Strain or plasmid	Description	Reference or source
*P. gingivalis* 33277		
Wild-type		(16)
YS19181	Δ*porX*::Em^R^	This work
YS19187	Δ*porY*::Em^R^	This work
YS19363	*porX-s*::Em^R^	This work
YS17814	Δ*sigH*::Em^R^	This work
YS18069	Δ*fimR*::Em^R^	This work
YS18131	Δ*rprY*::Em^R^	This work
YS18123	Δ*gppX*::Em^R^	This work
YS18345	Δ*PGN_0012*::Em^R^	This work
YS17819	Δ*PGN_0753*::Em^R^	This work
YS18343	Δ*PGN_0775*::Em^R^	This work
*P. gingivalis* W83		
Wild-type		(16)
YS19145	Δ*porX*::Em^R^	This work
YS19153	Δ*porY*::Em^R^	This work
*E. coli*		
DH5α	F^−^*sup*E44 Δ*lac*U169 (ϕ80 *lacZ* ΔM15) hsdR17 recA1 endA1 *gyrA*96 *thi*-1 *relA*1	Lab collection
BL21 (DE3)	F^−^*ompT hsdS*_B_ (r_B_^−^ m_B_^−^) *gal dcm* (DE3)	Lab collection
Plasmid		
pGEM-T-Easy	rep_pMB1, f1_ Amp^R^*lacZ*-*α*	Promega
pYS18019	rep_pMB1, f1_ Amp^R^*lacZ*-*α* del-*porY*	This work
pYS18485	rep_pMB1, f1_ Amp^R^*lacZ*-*α porX-s* (TAA)	This work
pYS17727	rep_pMB1, f1_ Amp^R^*lacZ*-*α del-sigH*	This work
pET21a	rep_ColE1_ Amp^R^*lacI* P_T7_	Novagen
pYS18456	rep_ColE1_ Amp^R^*lacI* P_T7_*porX (PGN_1019)*	This work
pGEM-*ermF*	rep_ColE1_ Amp^R^ Erm^R^*lacI* P_T7_	(47)
pT-COW	rep_ColE1_ rep_pB8-51_ Amp^R^ Cam^R^ Tc^R^	(48)
pYS18679	rep_ColE1_ rep_pB8-51_ Amp^R^ Cam^R^ Tc^R^	This work
	P_PGN_1016_*porX*_full-length_ (pT-COW-P_*PGN_1016*_-*porX*)	
pYS17713	rep_ColE1_ Amp^R^ Erm^R^*lacI* P_T7_*fimR*_3–305 nt_	This work
pYS17675	rep_ColE1_ Amp^R^ Erm^R^*lacI* P_T7_*rprY*_21–344 nt_	This work
pYS17692	rep_ColE1_ Amp^R^ Erm^R^*lacI* P_T7_*gppX*_33–572 nt_	This work
pYS17674	rep_ColE1_ Amp^R^ Erm^R^*lacI* P_T7_*PGN_0753*_14–320 nt_	This work
pYS17694	rep_ColE1_ Amp^R^ Erm^R^*lacI* P_T7_*PGN_00,12*_4–475 nt_	This work
pYS17673	rep_ColE1_ Amp^R^ Erm^R^*lacI* P_T7_*PGN_0775*_34–566 nt_	This work

### Plasmid construction

All plasmids used in this study are listed in Table 1. PCRs were performed using a Bio-Rad T100 Thermal Cycler with *Taq* DNA Polymerase (New England Biolabs). Custom oligonucleotides were synthesized by Integrated DNA Technologies (IDT) and listed in Table 2. PCR products were isolated using a QIAquick PCR purification Kit (QIAGEN). Restriction enzymes were purchased from New England Biolabs and used according to the manufacturer’s instructions. Digested DNA fragments were separated in 0.8 to 1% agarose gel electrophoresis and then isolated using a QIAquick Gel Extraction Kit (QIAGEN). Plasmids were purified from overnight cultures of *E. coli* DH5α in LB at 37 °C using Plasmid Mini Kit or Midi Kit (QIAGEN). Plasmid pYS18456 for production of PorX-c-His_6_ protein was constructed from plasmid pET21a digested with NdeI and XhoI and then ligated a NdeI- and XhoI-digested *porX* coding region, which was amplified with primers 2712 & 2713. A suicide plasmid normally contains an origin that initiates replication in one bacterial species, but not in another. When this plasmid integrates into the genomic DNA of the nonpermissive host, it will be replicated as a part of the chromosome. Plasmid pGEM-*ermF* (47) has a *Bacteroides* selection marker, the erythromycin resistance gene *emrF*, and carries an origin from ColE1, which is able to replicate in *E. coli*, but not in *P. gingivalis*. In this study, pGEM-*ermF* was used to construct the following suicide plasmids, each of which carried a DNA fragment from a specific *P. gingivalis* gene, thus allowing it to integrate into the target gene *via* a homologous recombination and disrupt the coding region. The resulting *P. gingivalis* mutants were selected for erythromycin resistance. Plasmid pYS18019 for mutagenizing the *porY* gene in 33277 and W83 strains was constructed from plasmid pGEM-*ermF* (47) digested with PstI and then ligated a PstI-digested fragment of a 26- to 526-nt *porY* coding region amplified by PCR using primers 2818 & 2819 and 33277 and W83 genomic DNAs as templates, respectively. Plasmid pYS18485 for constructing the *porX-s* strain was constructed using a fragment of a 19- to 669-nt *porX* coding region (a TAA codon was created by primer 3530), which was amplified with primers 2525 & 3530, digested with HincII, and then ligated with SmaI-digested pGEM-*ermF* plasmid. Plasmid pYS17727 for mutagenizing the *sigH* (*PGN_1740*) gene in 33277 strain was constructed using a fragment of a 3 to 244 nt *sigH* coding region, which was amplified with primers 1822 & 2937, digested with PstI, and then ligated with PstI-digested pGEM-*ermF* plasmid. Plasmid pYS18679 for complementation assay was constructed using PCR fragments containing the *PGN_1016* promoter region, which was amplified with primers 3464 & 3465, digested with HindIII and BamHI, and ligated between the HindIII and BamHI sites of a shuttle plasmid pT-COW (48); the resulting plasmid was digested with BamHI and XhoI, and then ligated with the *porX* coding region, which was amplified with primers 2951 & 2952 and digested with BamHI and XhoI. Plasmid pYS17713 for mutagenizing the *fimR* gene in 33277 strain was constructed using the 3- to 305-nt *fimR* coding region amplified by PCR with 33277 genomic DNA as template and primers 2752 & 2753, which was digested with PstI and ligated with PstI-digested pGEM-*ermF* plasmid. Plasmid pYS17675 for mutagenizing the *rprY* gene in 33277 strain was constructed using a 21 nt to 344 nt *rprY* coding region amplified with 33277 genomic DNA as template and primers 2756 & 2757, which was digested with PstI and ligated with PstI-digested pGEM-*ermF* plasmid. Plasmid pYS17692 for mutagenizing the *gppX* gene in 33277 strain was constructed using a 33 to 572 nt *gppX* coding region amplified with 33277 genomic DNA as template and primers 2758 & 2759, which was digested with PstI and ligated with PstI-digested pGEM-*ermF* plasmid. Plasmid pYS17674 for mutagenizing the *PGN_0753* gene in 33277 strain was constructed using a 14 to 320 nt *PGN_0753* coding region amplified with 33277 genomic DNA as template and primers 2758 & 2759, which was digested with PstI and ligated with PstI-digested pGEM-*ermF* plasmid. Plasmid pYS17694 for mutagenizing the *PGN_0012* gene in 33277 strains was constructed using a 4 to 475 nt *PGN_0012* coding region amplified with 33277 genomic DNA as template and primers 2765 & 2923, which was digested with PstI and ligated with PstI-digested pGEM-*ermF* plasmid. Plasmid pYS17673 for mutagenizing the *PGN_0775* gene in 33277 strains was constructed using a 34 to 566 nt *PGN_0775* coding region amplified with 33277 genomic DNA as template and primers 2767 & 2929, which was digested with PstI and ligated with PstI-digested pGEM-*ermF* plasmid. All plasmids were DNA sequenced before use.

**Table 2:** Primers used in this study^a^

Primer no.	Sequence
2166	cat gcc atg gaa aaa aac atg aga ccg
2167	ccg ctc gag tac ctt ttg ttt gaa aag atc c
2499	gga aga gaa gac cgt agc aca agg a
2500	gag tag gcg aaa cgt cca tca ggt c
2503	ata cac gtc cga cga tga gc
2504	cgc tga gca tgg att tca cg
2636	cgc cac tag gtt ctg atc
2637	gtc agg cgg caa ttg atg
2682	gat ccc aac agc gat cc
2683	atg gcc gcc gtt atc tc
2712	tat aca tat gga aaa aaa cat gag acc
2713	tat act cga gtc ttg ggt tgc atc gta att acg ggc
2752	aaa act gca gat tag tat cgt act cgt gg
2753	aaa act gca gcc ttc cac atc aat cgc c
2754	aaa act gca gtt atc gaa gat gaa ccg g
2755	aaa act gca gtc cag gag atg gaa tgg c
2756	aaa act gca gct ttc tct gcg agg acg
2757	aaa act gca gcg cat agc caa ctc ttc c
2758	aaa act gca gaa tgt cga ttt tgg tcg g
2759	aaa act gca gcc gag agg act gtc gag
2765	aaa act gca gaa agc ggc aat atc atc g
2767	aaa act gca gtg atg tgg cag tct gtg c
2818	aaa act gca gtc ttc gag cga agc cgc
2819	aaa act gca ggc tca gcc agc aag gcc
2822	aaa act gca gaa tag tgt gca att tcg c
2825	ccg ctc gag acc ttt tgt ttg aaa aga tcc
2923	aaa actgca gca gcc agc tcc aac acc tc
2929	aaa act gca gat cag cac aga ggc atg g
2951	cgc gga tcc tgc cgc ttc cgt tat acc c
2952	ccg ctc gag tta ctt ggg ttg cat cgt aat tac
2983	cgc gga tcc tta ctt ggg ttg cat cg
3043	tca tca gtc agc ttg tgg
3044	ccg agt acg ttt acc cc
3109	ttg ttt act ccg gtt cgg
3110	cca cga gga tgc agg gcc
3148	cat gcc atg gca atg agc agt ttc cac aag c
3149	ccg ctcg aga gcc gac atgc cca t
3247	ccg gaa ttc gat gtc aga caa cta cgg
3248	cgc gga tcc cca tag tac ggt ata cgg
3249	cgc gga tcc aag aga tgc ttg tgc ccg
3250	ccg gaa ttc ggt ttc ccc ttg aag agg
3269	ttc ttc gag cga agc cgc
3270	tct tgg tag cct gtg ccc
3463	ccg ctc gag tta tta gtg gtg gtg gtg gtg gtg ctt ggg ttg cat cgt aat tac
3464	ccc aag ctt tct ctt tga ata ctt gcg gc
3465	cgc gga tcc ctt gcg gaa att ata cag g
3525	cag gtc gac ccg tat acc gta cta tgg
3530	cag gtc gac tta tac ctt ttg ttt gaa aag atc
3568	cta gct agc tta tac ctt ttg ttt gaa aag atc
3601	cgc gga tcc gca aca tta aac ctg ccg
3837	atg tag gga tgc atg ccc
3838	caa agt cgg aag caa acg
3912	gtc agt tct tcc act cgg
3913	gga aga atg gtc aga tcg
4025	aga gag cga ctc tca acg
4026	cac acg ttc tat att gcg
4105	cag gcg ctc ggt tcc gcg ttt ttc ttt gca ata ag
4106	cgc gga tcc cag cgc ctg aaa cag aag caa c
4344	tcc tta tcc atg cga ttg
4345	ggg tat ctt taa tcg ggc

a All oligonucleotides were purchased from IDT (Integrated DNA Technologies).

### Construction of strains with chromosomal mutations

*P. gingivalis* mutants or other genetic engineered strains were constructed by introducing plasmids or DNA fragments into recipient cells using an electroporation procedure suggested previously (16). The Δ*porX* mutants of 33277 and W83 strains were constructed by replacing the *porX* gene with the erythromycin resistance cassette (Em^R^). DNA regions from the upstream and downstream of the *porX* coding region were amplified by PCR from the 33277 genome using pairs of primers, 3247 & 3248 and 3249 & 3250, respectively. The amplified upstream and downstream DNAs were digested with BamHI, respectively, and were ligated together. The ligated product was digested with EcoRI and ligated with the EcoRI-digested pGEM-T-easy vector. Plasmid pGEM-*ermF* was digested with BamHI, and the 1.1-kb *ermF* DNA fragment was isolated and inserted into the BamHI site located between the *porX* upstream and downstream regions carried in the resulting plasmids to yield plasmid for mutagenesis. This plasmid was used as the template for PCR amplification with primers 3247 & 3250, and the PCR product was introduced into 33277 and W83 wild-type strains by electroporation to get the YS19181 and YS19145 strains, respectively. The Δ*fimR*, Δ*rprY*, Δ*gppX*, Δ*PGN_0753*, Δ*PGN_0012*, Δ*PGN_0775*, Δ*porY*, and Δ*sigH* mutants ware constructed by introducing suicide plasmids pYS17713, pYS17675, pYS17692, pYS17674, pYS17694, pYS17673, pYS17409, and pYS17727 into the 33277 wild-type strain, respectively. The *porX-s* strain was constructed by introducing suicide plasmid pYS18485 into the 33277 wild-type strain. Mutated sequences in these strains were confirmed by DNA sequencing.

### Quantitative real-time PCR

Bacterial cells were grown anaerobically in BHI medium at 37 °C for 48 h. Total RNAs were isolated from bacterial cultures using a High Pure RNA Isolation Kit (Roche) according to the manufacturer’s instructions. The concentration of RNA samples was determined by measuring absorbance at 260 nm using a spectrophotometer (SmartSpec Plus, BIO-RAD). The quality of RNAs was evaluated in a 1.2% agarose gel electrophoresis. cDNAs were synthesized using random primers (IDT) and a murine leukemia virus reverse transcriptase (NEB). The amount of cDNA was quantified using PowerUp SYBR Green Master Mix with primers 3837 & 3838 for *porT*, 3912 & 3913 for *PGN_0341*, 2503 & 2504 for *porX*, 3269 & 3270 for *porY*, 2682 & 2683 for *sigH*, and 2499 & 2500 for *rpoB* (Table 2), and qPCR was performed in QuantStudio 3 Real-time PCR Systems (Applied Biosys, Thermo Fisher Sci).

### Isolation of the full-length PorX-c-His_6_ protein

*E. coli* BL21-Gold (DE3) harboring plasmid pYS15493 (pET15b-*porX-His*_*6*_) was grown in 500 ml of LB medium by shaking at 37 °C to OD_600 nm_ 0.5; then IPTG was added to a final concentration of 0.4 mM, and bacterial cells were cultured for another 2 h. Bacterial cells were harvested by centrifuging at 10,000*g* for 15 min and washed with 50 ml of PBS once, suspended in 10 ml of PBS, and opened by sonication (Misonix Sonicator 3000). The cell lysate was used for purification of the PorX-c-His_6_ protein with a Ni-NTA Affinity Gel (QIAGEN) by following the instructions from the manufacturer. The purity and concentrations of protein samples were tested using Silver Staining Kit (Pierce) and BCA Protein Assay Kit (Pierce) by following the instructions from the manufacturer.

### Primer extension

This assay was performed as described (49) with the following modifications. Total RNAs were isolated from *P. gingivalis* cultures using the High Pure RNA Isolation Kit (Roche) according to the manufacturer’s instructions. Primer 4025 was labeled with [γ-^32^P] ATP (PerkinElmer) using T4 polynucleotide kinase (NEB). Primer extension was performed in a 20-μl mixture with 10 μg of RNA, 2 μl of ^32^P-labeled primer 4025, and 25 units of M-MuLV reverse transcriptase (NEB) at 42 °C for 2 h. Synthesized cDNAs were precipitated with 2.5 volumes of ethanol and 0.3 M NaAc and resuspended in 5 μl of Gel Loading Buffer II (Thermo Fisher). cDNA samples were analyzed by a 6% denaturing polyacrylamide gel with a DNA ladder amplified from the chromosome with primers 3044 and ^32^P-labeled 3043 and generated by Maxam–Gilbert reaction.

### Electrophoretic mobility shift assay (EMSA)

The EMSA was performed as described (49, 50) with the following modifications. Primer 3043 was labeled using T4 polynucleotide kinase (New England Biolabs) and [γ-^32^P]-ATP (PerkinElmer Life Sciences). The *porT* promoter region was amplified by PCR using chromosomal DNA as a template with primers ^32^P-4025 & 4026. In total, 0.5 pmol of ^32^P-labeled DNA fragments was incubated at room temperature for 30 min with 0, 50, or 100 pmol of PorX-c-His_6_ protein in 20 μl of an EMSA buffer consisting of 20 mM HEPES-KOH (pH 7.9), 20% glycerol (vol/vol), 0.2 mM EDTA, 0.1 M KCl, 0.5 mM phenyl-methylsulfonyl fluoride (PMSF), 1 mM DTT, Poly (dI:dC) (0.02 μg/μl), BSA (1.25 μg/μl), 7.5 mM MgCl_2_. In total, 5 pmol of unlabeled DNA (namely cold) was added to ^32^P-labeled DNA when required. After the addition of the DNA dye solution (40% glycerol, 0.05% bromophenol blue, 0.05% xylene cyanol), the mixture was directly subjected to 5% polyacrylamide electrophoresis. Signals were detected by autoradiography.

### DNase I footprinting analysis

This assay was performed as described (49) with the following modifications. Primer 4025 was labeled with T4 polynucleotide kinase (NEB) and [γ-^32^P] ATP (PerkinElmer). The *porT* promoter region was amplified by PCR using chromosomal DNA as a template with primers ^32^P-4025 & 4026. For DNase I footprinting assay, approximately 25 pmol of the ^32^P-labeled DNA and 0, 70, 140, or 280 pmol of the PorX-c-His_6_ protein were mixed in a 100 μl reaction. DNase I digestion was carried out as described previously (51) using 0.05 units DNase I (Invitrogen) per reaction. Samples were analyzed by 6% denaturing polyacrylamide electrophoresis by comparison with a DNA sequence ladder generated by Maxam and Gilbert A + G reaction, using the same ^32^P-labeled PCR product. The positions of radioactive DNA fragments in the gels were detected by autoradiography.

### H_2_O_2_ sensitivity assay

The assay was performed as described (46, 52) with the following modifications. Overnight cultures of the 33277 strains grown in BHI at 37 °C were used to inoculate in BHI to OD_600nm_ 0.2. When the OD_600nm_ of the cultures doubled, each culture was split into two tubes (3 ml each), and one was treated with 0.5 mM H_2_O_2_ (final concentration) and the other was left untreated to serve as a control. All cultures were incubated at 37 °C for 24 h and 1 ml of each culture was used to measure the OD_600nm_ (SmartSpec Plus, BIO-RAD).

### Virulence assay in a mouse model

All animal experiments conform to our animal protocols (18-1655R) approved by the Institutional Animal Care and Use Committee (IACUC), Office of Research Integrity and Assurance, Arizona State University (ASU Protocol Number: 18-1655R). Groups of 6-week-old female BALB/c mice (purchased from Charles River Laboratories) were randomly allocated into different groups. Determination of virulence of the *P. gingivalis* W83 and mutant strains was performed using mouse subcutaneous infection experiments, as described previously (53) with slight modifications. Briefly, bacterial cells were grown in the enriched BHI broth at 37 °C for 12 h. The culture was diluted 20 times in 100 ml of fresh BHI medium and grew for a period as indicated. The cells were harvested by centrifuging at 10,000*g* for 20 min and washed once with PBS, then adjusted to 5 × 10^11^ CFU/ml in PBS. If needed, other concentrations of bacterial cells were used. Mice were challenged with subcutaneous injections of 0.1 ml at each of the two sites on the depilated dorsal surface (0.2 ml per mouse). Infected mice were examined daily for survival. Three sets of experiments were carried out. The median lethal dose (LD_50_) was calculated with a formula described previously (45).

### Coculturing dendritic cell with *P. gingivalis* W83 lysate

Mouse bone-marrow-derived dendritic cells (BMDCs) were isolated from femurs and tibias and cultured in the complete RPMI-1640 medium containing mouse GM-CSF (20 ng/ml), mouse IL-4 (10 ng/ml), and 2-mercaptoethanol (55 μmol) for 7 days according to a previous study (54). Then 3 × 10^4^ per well BMDC were seed into 96-well plate in complete RPMI-1640 medium (without GM-CSF and IL-4). After 2 days, 0.1 μg per well bacterial lysate (W83) was added to BMDCs for 16 h for cytokine release assay.

### Coculturing T cell with *P. gingivalis* W83 lysate

Mouse splenocytes were initially plated into 6-well plates (5 × 10^6^ per well) in RPMI medium (Hyclone) containing 10% heat-inactivated fetal bovine serum (Sigma) and supplemented to a final concentration with IL-7 (0.5 ng/ml recombinant mouse, R & D Systems) and IL-2 (30 U/ml), l-glutamine (2 mM), penicillin (50 U/ml), streptomycin (50 μg/ml), 2-mercaptoethanol (50 μM), transferrin (68.8 μM), and sodium selenite (3.9 nM), all from Life Technologies. Then T cells were activated with Dynabeads Mouse T-Activator (Thermo Fisher) for 24 h. Then 5 × 10^4^ per well T cells were seed into 96-well plate in the complete RPMI-1640 medium. After 2 days, 0.1 μg per well bacterial lysate (W83) was added to T cells for 16 h for cytokine release assay.

### Multiplexing immunoassay

The culture supernatants (25 μl) were collected after 16 h of coculture for analysis of secreted cytokine concentrations. IFN-γ, IL-6, IL10, and IL-1β were multiplexed using the ProcartaPlex Mouse custom kit (eBioscience) using undiluted supernatant and analyzed using the Magpix system (Thermo Fisher).

### Flow cytometry

Splenocytes were harvested, and the single-cell suspension was prepared. PE-CF594 anti-mouse CD3e and FITC anti-mouse CD8a were purchased from BD Biosciences; PE-anti-mouse CD4 antibody, PE-Cy7 anti-mouse CD8a antibody, FITC-anti-CD25 antibody, and PE anti-FoxP3 antibody were purchased from Thermo Fisher. Cells were resuspended in 2% BSA/PBS and stained with fluorochrome-conjugated antibodies at room temperature for 30 min. The cells were washed twice in 2% BSA/PBS and fixed in 1% paraformaldehyde before fluorescence-activated cell sorting (FACS) analysis. All samples were analyzed using the Attune NxT Flow Cytometer (Thermo Fisher). Data analysis was performed using the FlowJo 10 software (Treestar).

## Data availability

All of the data is contained within the article.

## Supporting information

This article contains supporting information.

## Conflict of interest

The authors declare that they have no conflict of interest.
