# Supplementary material for: The PorX/PorY system is a virulence factor of Porphyromonas gingivalis and mediates the activation of the type IX secretion system
Source: J Biol Chem. 2021 Mar 21;296:100574. doi: 10.1016/j.jbc.2021.100574 (PMC8050853; doi:10.1016/j.jbc.2021.100574)
Supplement: Supplemental Figures S1–S3 [file mmc1.pdf]

The PorX/PorY system is a virulence factor of *Porphyromonas gingivalis* and mediates the activation of the Type IX secretion system

Dezhi Yang[1,2], Chizhou Jiang[1,2], Bo Ning[3], Wei Kong[2*], and Yixin Shi[1,2*]

[1]The School of Life Sciences, and [2]Biodesign Center for Immunotherapy, Vaccines and Virotherapy, Arizona State University, 85287-4501, [3]The Center for Cellular and Molecular Diagnostics, Tulane University School of Medicine, 70112

[*]Correspondence should be addressed to:
Wei Kong, wei.kong@asu.edu or Yixin Shi, yixin.shi@asu.edu

Running Title: Gene regulation of the PorX/PorY system in *P. gingivalis*

This file includes:
Figure S1 to S3

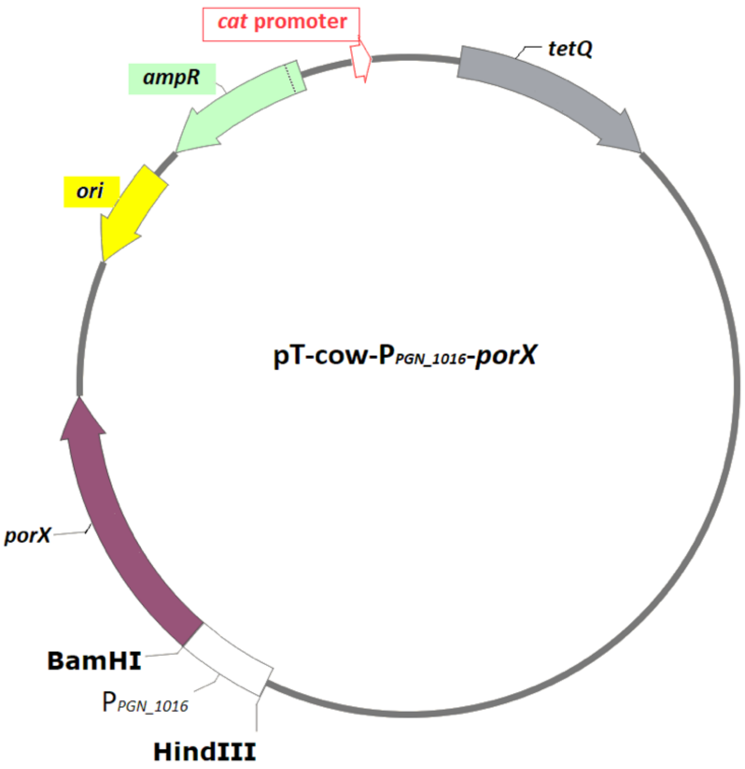

Fig. S1

Fig. S1. Map of the *porX* complementation plasmid. The plasmid pT-COW-P$_{PGN_1016}$-*porX* contained a wild-type copy of the *porX* coding region (marked as the *purple curved arrow*). Transcription of this gene was initiated by a promoter located upstream of the *PGN_1016* gene. Both *PGN_1016* and *porX* genes were predicted to be members of an operon on *P. gingivalis* genomes.

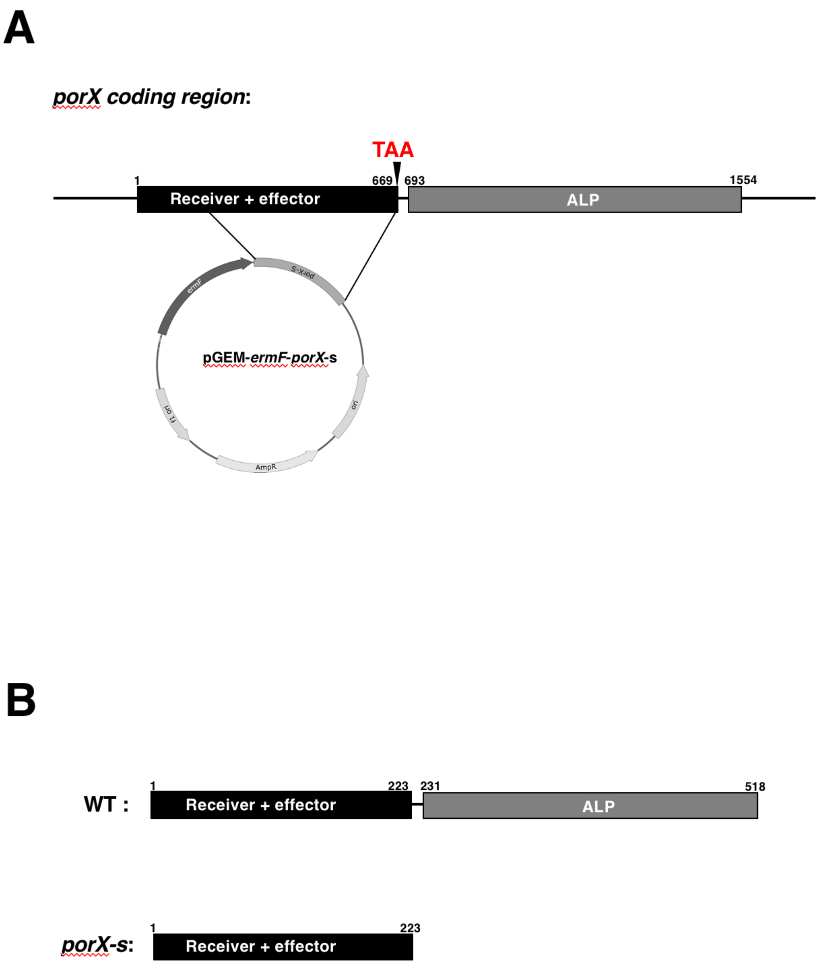

Fig. S2

F

Fig. S2. Schematic representatives of the strategy to construct the *porX-s* strain. (A) The *porX* coding region. The *black box* represents the DNA region encoding the first 223 amino acid residues with receiver + DNA-binding regions, and the *grey box* represents the DNA region encoding the ALP region. Suicide plasmid pGEM-*ermF-porX-s* was inserted into the *porX* coding region to introduce a TAA stop codon between the 669th-670th nucleotides of the *porX* coding sequence (indicated by an *arrowhead*). *Numbering* represents the nucleotides of the coding sequence. **(B)** Structures of the PorX proteins. The full-length wild-type PorX protein (*upper*) and truncated PorX-s protein (*bottom*). The first 223 amino acid residues (*black box*) represent receiver + DNA-binding regions shared by both WT PorX and PorX-s proteins. The ALP sequence (*grey box*) was deleted in PorX-s protein. *Numbering* represents codon position.

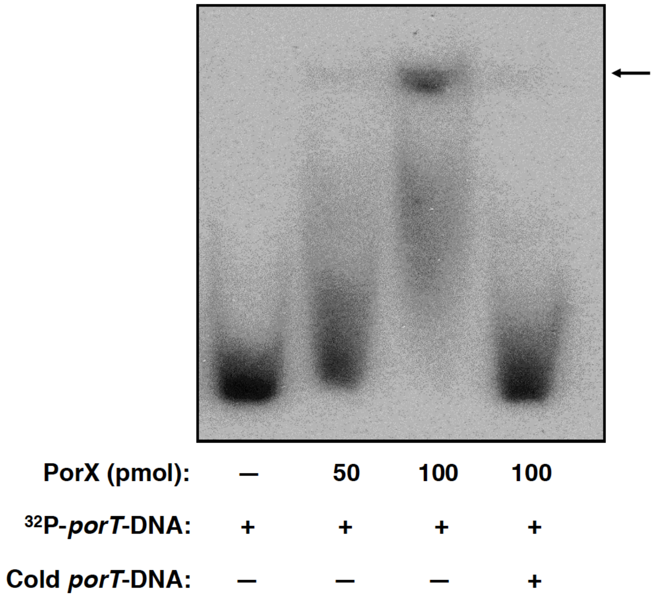

Fig. S3

Fig. S2. The PorX response regulator binds to the *porT* promoter region. EMSA analysis for binding of PorX to the *porT* promoter. ^{32}P-labeled *porT* DNA fragment was incubated with PorX-c-His_6 protein at the indicated amount. The 4th lane is the same as the 3rd lane but supplemented with non-labeled (cold) *sigP* DNA fragment. The PorX/DNA mixtures were subjected to 5% PAGE. Location of DNA migration was detected by autoradiography. *Arrow* indicates the shifted bands after DNA fragments were associated with the PorX-His_6 protein.
